# Multirate method for co-simulation of electrical-chemical systems in multiscale modeling

**DOI:** 10.1007/s10827-017-0639-7

**Published:** 2017-04-07

**Authors:** Ekaterina Brocke, Mikael Djurfeldt, Upinder S. Bhalla, Jeanette Hellgren Kotaleski, Michael Hanke

**Affiliations:** 1 Science for Life Laboratory, School of Computer Science and Communication, KTH Royal Institute of Technology, Stockholm, Sweden; 2 National Centre for Biological Sciences, Bangalore, India; 3 Manipal University, Manipal, India; 4 PDC Center for High-Performance Computing, KTH Royal Institute of Technology, Stockholm, Sweden; 5 Department of Neuroscience, Karolinska Institute, Stockholm, Sweden; 6 Department of Mathematics, School of Engineering Sciences, KTH Royal Institute of Technology, Stockholm, Sweden

**Keywords:** Multiscale modeling, Multiscale simulation, Co-simulation, Coupled system, Multirate integration, Adaptive time step integration, Backward differentiation formula, Parallel numerical integration, Coupled integration

## Abstract

Multiscale modeling by means of co-simulation is a powerful tool to address many vital questions in neuroscience. It can for example be applied in the study of the process of learning and memory formation in the brain. At the same time the co-simulation technique makes it possible to take advantage of interoperability between existing tools and multi-physics models as well as distributed computing. However, the theoretical basis for multiscale modeling is not sufficiently understood. There is, for example, a need of efficient and accurate numerical methods for time integration. When time constants of model components are different by several orders of magnitude, individual dynamics and mathematical definitions of each component all together impose stability, accuracy and efficiency challenges for the time integrator. Following our numerical investigations in Brocke et al. (*Frontiers in Computational Neuroscience, 10*, 97, 2016), we present a new multirate algorithm that allows us to handle each component of a large system with a step size appropriate to its time scale. We take care of error estimates in a recursive manner allowing individual components to follow their discretization time course while keeping numerical error within acceptable bounds. The method is developed with an ultimate goal of minimizing the communication between the components. Thus it is especially suitable for co-simulations. Our preliminary results support our confidence that the multirate approach can be used in the class of problems we are interested in. We show that the dynamics ofa communication signal as well as an appropriate choice of the discretization order between system components may have a significant impact on the accuracy of the coupled simulation. Although, the ideas presented in the paper have only been tested on a single model, it is likely that they can be applied to other problems without loss of generality. We believe that this work may significantly contribute to the establishment of a firm theoretical basis and to the development of an efficient computational framework for multiscale modeling and simulations.

**Electronic Supplementary Material:**

The online version of this article (doi:10.1007/s10827-017-0639-7) contains supplementary material, which is available to authorized users.

## Introduction

Multiscale modeling and simulations provide an entirely new capability for scientists to explore complex brain phenomena in neuroscience (Bhalla 2014; Hirakis et al. 2015). Multiscale modeling may contribute to understanding of how the hierarchy of biological levels (Djurfeldt and Lansner 2007) integrates to produce certain brain functions. This knowledge may have a significant impact for elucidation of disease states and drug development.

In the paper we refer to multiscale modeling as the process of defining multiple models on the conceptual, mathematical and computational levels (Delalondre et al. 2010) as well as their assembly into a larger model. Our ultimate goal is to be able to simulate these models simultaneously, in different simulation tools, while exchanging data during runtime. We refer to this methodology as co-simulation (Park and Felippa 1983; Felippa et al. 2001). In computational neuroscience the co-simulation idea is used by several software tools. For instance, the MUlti-SImulation Coordinator (MUSIC) tool promotes interoperability between different event based simulators by allowing existing models defined by different formalisms to be simulated in parallel (Djurfeldt et al. 2010).

In computational neuroscience the mathematical domain is usually represented by a particular set of mathematical formalisms among which are systems of Ordinary Differential Equations (ODEs). During the co-simulation of mutually dependent systems the coupling of the models described by different formalisms or even the same mathematical formalism is not a trivial task. One of the problems the scientist may observe is numerical artifacts such as numerical instability as well as inaccuracy. Thus the potential of a co-simulation methodology depends on the coupling method between the components, the choice of an appropriate organization strategy and the approximation of the exchange variables (Brocke et al. 2016). Moreover, the co-simulation technique imposes additional restrictions. In order to minimize the communication overhead during a simulation the exchange of data should be limited to certain points in time; an intermediate communication is prohibited.

Brocke et al. (2016) present an efficient method for coupling problems that can be formulated by systems of Ordinary Differential Equations (ODEs). In order to cope with numerical stiffness the method implements the decoupled Backward Differentiation Formulae (BDF). The integration step size is predicted by the adaptive controller in Söderlind and Wang (2006). The controller is based on the error estimation mechanism proposed and analyzed in Skelboe (2000). Brocke et al. (2016) show a significant advantage of the method both with respect to accuracy and computational cost while simulating the electrical-chemical test case. The method uses a *singlerate* coupling concept, that is it works with step sizes that are varying in time but are the same for each component per step. In contrast, in a *multirate* approach each component can be solved with its own discretization time step (Gear and Wells 1984). This can reduce the number of communication points between the components, facilitate the throughput and gain computation efficiency during co-simulation.

Skelboe (2000) mentions that the decoupled integration formula can be used in a multirate mode with a waveform relaxation method. Waveform relaxation is an iterative method. It is an expensive method and suitable only for a certain class of problems (Sand and Skelboe 1992). Sometimes it is enough to have only one iteration during the relaxation but this is only possible if a macro step size (i.e. the time step between two successive communication points) is sufficiently small. To the best knowledge of the authors, the choice of multirate methods applicable to the physical systems of our interest is limited. Bartel and Günther (2002) propose a multirate method suitable for highly integrated electric circuits that usually exhibit the variables of different time scales. This integration approach employs the concept of a constant step size integration over a varying in time macro time step. However, the results showed a relatively high amount of rejected macro steps for some stiff test cases. Another multirate method for a system of ODEs is discussed in Engstler and Lubich (1997). The method belongs to a class of extrapolation methods in which the reduction of the number of function evaluations is achieved by intermediate interpolations. These methods require additional communication between the components that can become expensive in co-simulations and should be avoided. Similar ideas are presented in Savcenco (2008).

Brocke et al. (2016) design the method mathematically justified for a singlerate approach. Here we try to relax the requirement of a singlerate integration by applying the idea of a multirate integration to gain more efficiency during co-simulations of electrical-chemical systems. The presented algorithm defines a sequence of actions to be taken while simulating mutually dependent components of a multiscale system in parallel. In particular, it introduces a new strategy for the error control in the multirate integration approach. The algorithm allows each component to be solved independently during the macro time step while keeping the accuracy withing acceptable bounds. One of the goals of the algorithm is to minimize communication between the components during a simulation. Thus it is especially tailored to suit co-simulations.

In the Section 2 we address the questions “which communication signal to choose?”, “when do the components have to communicate or to be synchronized?”, “in which order can the components of a multiscale system be solved?”. Then we present the multirate algorithm and describe the details of its implementation in the Section 2.2. In the Sections 2.3 and 2.4 we describe the multiscale test case and an evaluation technique, respectively. Finally, we present and discuss the results in the Sections 3 and 4 sections.

## Materials and methods

### Problem definition

For expository purposes we define the problem as a system of ODEs:

1 $$\begin{array}{@{}rcl@{}} \frac{d}{dt}x_{1} &=& f_{1}(t,x_{1},g_{1}(x_{2}))\\ \frac{d}{dt}x_{2} &=& f_{2}(t,g_{2}(x_{1}),x_{2}), \end{array} $$

where *x*_1_,*x*_2_ are solution vectors of the component *1* and the component *2* respectively. *g*_1_ and *g*_2_ are transformation functions of the respective solution vector (or a part of it). In the simplest case the transformation function may represent a uniform scaling operation between the variables.

#### Communication signal

Coupling the components of a multiscale system involves decisions about communication signals. The choice can be crucial not only from the conceptual perspective, but it may also have a significant impact on the computational side of a simulation, e.g., with respect to efficiency.

In neuroscience, the cellular level is a level of communications, signal integration and filtering that usually acts on a timescale of about a few microseconds. In the context of synaptic plasticity, learning and memory, the subcellular level covers long term processes of physiological changes in the cell that can span timescales from a few seconds to months and years. How does the dynamics of a communication signal contribute to the numerical properties and overall simulation performance? Which time course should a communication signal follow in this case?

In Brocke et al. (2016) we developed a multiscale test case where the fast electrical signal is scaled and communicated to the biochemical component. From a computational perspective, the fast changing signal can introduce stiffness in the whole coupled system (Gear and Wells 1984). However, if stiffness is isolated in a separate component, it can be tackled locally by choosing an appropriate integration method. Consequently the choice of having an approximated communication signal with time course of a slower component seems to be more favorable unless there is a need to model systems on a very small spatial scale.

#### Organization of system components

In co-simulations the components can be solved using either *Jacobi* or *Gauss-Seidel* organization between the components (Brocke et al. 2016). The organization of the components will define which approximation, interpolation, extrapolation, or both, shall be used. The choice may have a crucial impact on the accuracy of the coupled simulation. In general, during a multirate integration when approximation is required, it is preferable to choose interpolation instead of extrapolation over a large integration step. Gauss-Seidel organization allows us to eliminate the error introduced by the approximation of one component as described in Brocke et al. (2016). Therefore, we will consider only Gauss-Seidel organization with the fast component or the slow component solved first. We will refer to these as fast-first and slow-first strategies respectively.

**Fig. 1:**
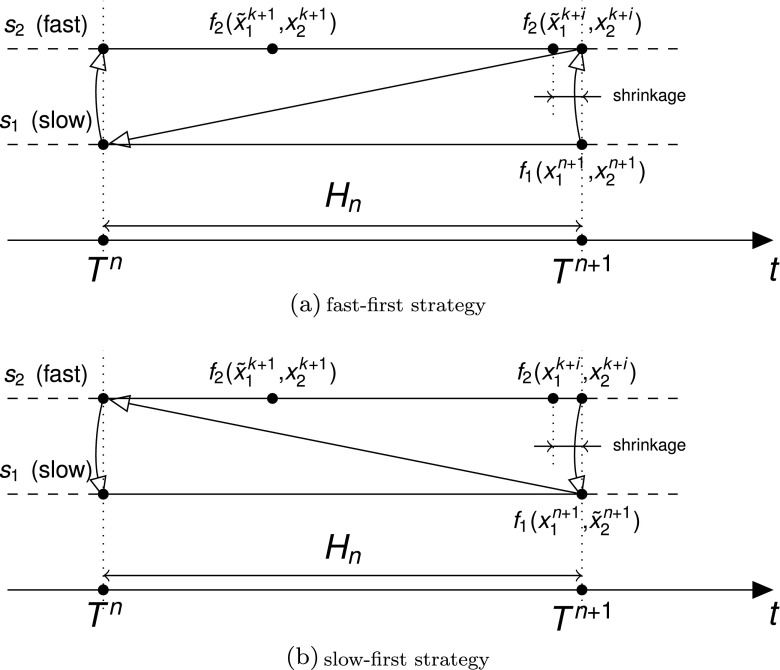
Discretization of two components *s*_1_ and *s*_2_ over one macro time step [*T* ^*n*^,*T* ^*n*+1^]. f1(...) and f2(...) are the function evaluations required to evolve the system. The *arrows* correspond to the communication of exchanged variables. The variables marked with a *tilde* denote variables obtained either by extrapolation or interpolation. In the fast-first strategy (1a) the slow variables ${x^{n}_{1}}$ are communicated first, then for each micro time step the variables $\tilde x^{k+i}_{1}$ are obtained by extrapolation ${x^{n}_{1}}$ at each $t^{k+i}_{2}$. The calculated solution $x^{k+i}_{2}$ at *T* ^*n*+1^ is communicated backwards. Finally, the slow component evolves to *T* ^*n*+1^. In the slow-first strategy (1b) the sequence of computations is similar. The difference is in how the exchanged variables of the slow component are approximated at each micro time step. $\tilde x^{k+i}_{1}$ can be obtained by interpolation since it is the slow component that makes the first step. Both figures show a synchronous communication between the components where the last micro time step $[t^{k+i-1}_{2},t^{k+i}_{2}]$ is shrunk (see the discussion in Section 2.1.3). This interval is marked with *two arrows* and the label “shrinkage”

Figure 1 shows the discretization of two components over one macro time step *H*_*n*_ for the fast-first (Fig. 1a) and slow-first (Fig. 1b) strategies. The slow system values $x_{1}^{k+l}$, needed at each micro time step *k* + 1…*k* + *i*, are not known. Then depending on the strategy either an extrapolation or interpolation can be used to calculate $\tilde x^{k+1}_{1}$.

Each strategy has its pros and cons. In the fast-first strategy when a macro time step is rejected at least some of the computations performed at the micro time steps will be discarded. In this case it is crucial to use a well adjusted step size controller in order to minimize the number of rejected steps. In contrast, when a slow component is solved first the memory usage and computation costs are relatively negligible. However, an extrapolation over a large macro step can introduce a significant extrapolation error. In principle, the error can be mitigated when the coupling from a fast component to a slow component is weak (Sand and Skelboe 1992). We expect that this can be accomplished by using a slow changing communication signal in our test case.

#### Communication time points

**Fig. 2:**
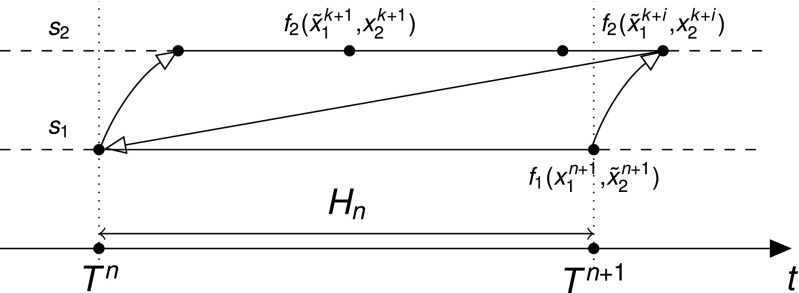
Discretization of two components *s*_1_ and *s*_2_ with asynchronous communication over one macro time step [*T* ^*n*^,*T* ^*n*+1^]. The *arrows* correspond to the communication of exchanged variables in the fast-first integration strategy. The variables marked with a *tilde* denote variables obtained by extrapolation. Asynchronous communication requires an interpolation of $x^{k+i}_{2}$ at *T* ^*n*+1^ that is denoted by $\tilde {x}^{n+1}_{2}$ in the figure

Co-simulation usually imposes the problem of the variables’ availability in the mutually dependent components. Moreover, in a multirate integration approach there will be points on the time grid when not all required variables are available due to the independent discretization of the components. With *synchronous communication* we refer to a communication protocol organized in a such way that the time points of the discretization grid of any component is a subset of the grids of the faster components. In contrast, in *asynchronous* communication, the discretization time grids of the components are not synchronized as depicted in Fig. 2. It is worth noting that the latter type of communication requires an additional interpolation step.

Several tactics are known for synchronous communication. For instance, one strategy is to use a fixed step size ratio between the micro and macro time steps (Bartel and Günther 2002). One of the potential problems with this approach is a relatively high amount of rejected macro time steps. Another option can be to shrink the last micro time step at the end of each macro time step as indicated in Fig. 1. However, a sharp reduction of the step size can cause inefficiency of the step size controller that will require a number of additional small steps to be taken before the step size will be increased again. Moreover, it was shown that the BDF method has better stability properties with a smoother behavior of step sizes (Hairer et al. 2010, pp. 402ff). The latter was the key argument that inclined us towards the choice of asynchronous communication.

### Multirate algorithm

Here we give a detailed description of the multirate algorithm. Let *T* be an integration interval and *S* an ordered set of *p* components of a system to be integrated over *T*. Then the system can be solved by the SOLVE_INTERVAL(T,S) function. A sequence of necessary operations is presented in Algorithm 1 together with the following explanations:

1. The algorithm admits a possible variance in the temporal dynamics of a component that may appear due to an external signal, for instance. The defined set *S* has the order slowest to fastest. A fast component is the one with the smallest predicted integration time step while a slow component is the one with the largest. Thus, the decision which component is fast and which is slow can be made automatically during the integration process. The order of components can be assigned by a simple algorithm like *insertion sort* before each call to the SOLVE_INTERVAL(T,S) function.
  In the problem definition Eq. (1) we have two components *p* = 2, slow *s*_1_ and fast *s*_2_, respectively. Note, that the notation *f*_*i*_ introduced in Eq. (1) denotes the physics of a multiscale system while *s*_*i*_ is used to distinguish between fast and slow components in the algorithm.
2. For each current component *s*_*r*_ an integration time interval *T* is provided by the consecutive slower component. For the outermost call of SOLVE_INTERVAL the variable *T* is equal to the next predicted time step of the first component in the set *S*, that is the largest predicted time step among all components.
3. Different numerical approximation methods could be applied to each component to handle individual dynamics in an efficient way. Here, we choose to use one method for all components in the system. We implement the Backward Differential Formulae of the second order (BDF2) due to its good stability properties, high applicability for stiff differential equations and analyzed properties in coupled simulations (referred to as decoupled BDF2 formulae in Skelboe (2000)). The SOLVE function calculates a numerical solution at $t^{n+1}_{r}$ of the current component *s*_*r*_ using the BDF2 method (see Section 2.2.1 for details).
4. A set of exchanged variables $\{\tilde {x}^{n+1}_{1},\ldots ,\tilde {x}^{n+1}_{q}\}$ of the components {*s*_1_,…,*s*_*q*_} at time *t* ^*n*+1^ has to be provided to the SOLVE function.
  In the slowest-first strategy the subset $\{\tilde {x}^{n+1}_{1},{\ldots } ,\tilde {x}^{n+1}_{r-1}\}$ is obtained by interpolation at $t^{n+1}_{r}$; while the subset $\{\tilde {x}^{n+1}_{r+1},{\ldots } ,\tilde {x}^{n+1}_{q}\}$ is obtained by extrapolation at $t^{n+1}_{r}$.
  In the fastest first strategy with synchronous communication between the components only extrapolation is required to obtain the $\{\tilde {x}^{n+1}_{1},{\ldots } ,\tilde {x}^{n+1}_{r-1}\}$ subset since the exchanged variables from the faster components are calculated first. In asynchronous communication between the components an interpolation can be used to calculate the subset $\{\tilde {x}^{n+1}_{r+1},{\ldots } ,\tilde {x}^{n+1}_{q}\}$.
  Note that for notational simplicity, we omitted the transformation function *g* that was introduced in Eq. (1). The exchanged variables usually represent a result of the transformation function *g* that can be as simple as a subset of the variables of a solution vector *x*.
5. An estimation of the local discretization error $|\left [\epsilon _{r}\right ]|$ is calculated in the ESTIMATE_ERROR function (line #9). Each component calculates an estimation of the local discretization error using a second order polynomial predictor (Brocke et al. 2016). More details are given below in the Section 2.2.2 section.
6. The PREDICT function implements a step size controller that predicts an optimal next time step. We use the step size controller implemented in Brocke et al. (2016).

**Figure:**
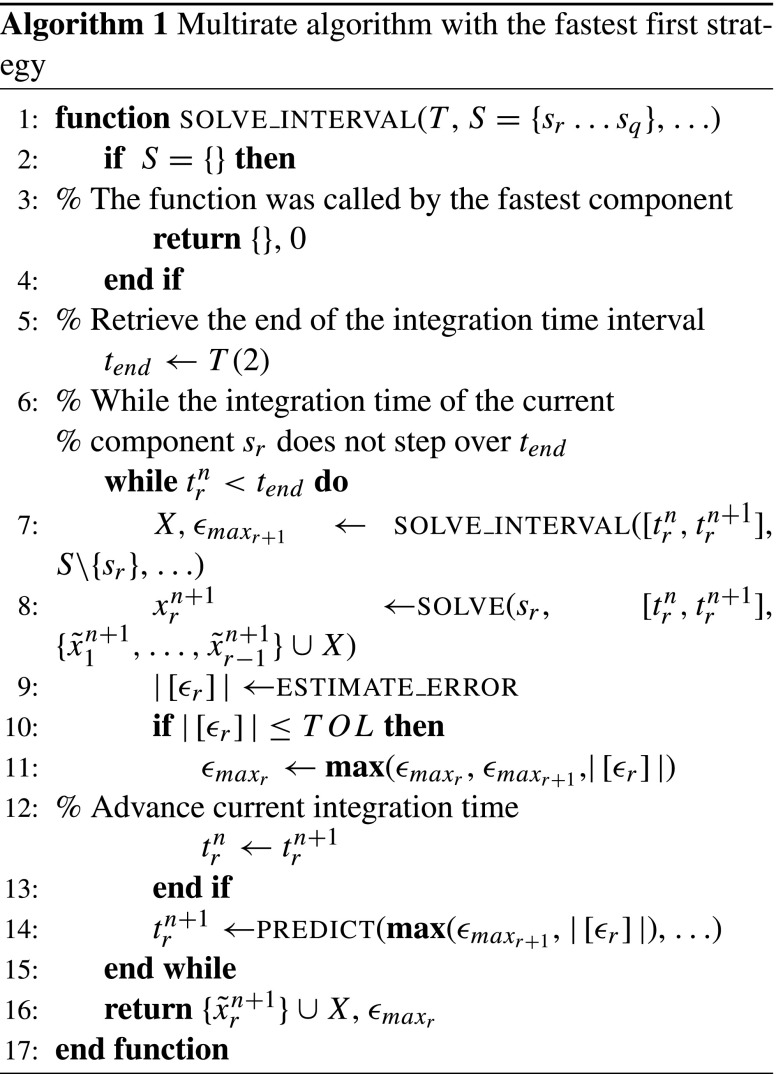

**Fig. 3:**
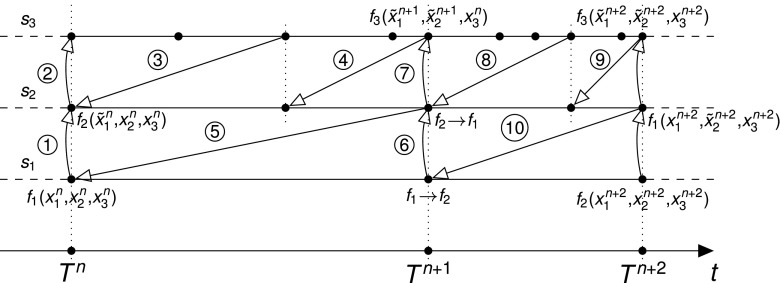
The sequence of communications realized by the fast-first integration strategy with synchronous communication between three components *s*_1_, *s*_2_ and *s*_3_ over two macro time steps [*T* ^*n*^,*T* ^*n*+1^] and [*T* ^*n*+1^,*T* ^*n*+2^]. The arrows correspond to the communication of exchanged variables. The order of communications is denoted by the circled numbers. After the first macro time step the second subsystem was recognized as the slowest component. Thus the order of integration has been changed (denoted with $f_{1}\rightarrow f_{2}$ and *f*_2_ → *f*_1_, respectively)

Figure 3 shows an example of the communication pattern between three components *s*_1_, *s*_2_ and *s*_3_ implemented by the Algorithm 1. The algorithm assumes an all-to-all connectivity between the components in the system. An optimization for non all-to-all cases has yet to be considered.

#### Backward differentiation formula methods

Here we are addressing a class of problems that is described by a system of ODEs with a given initial value (initial value problem (IVP)). This class of problems is usually solved by finite difference time-stepping methods. We use the Backward differentiation Formula (BDF) methods. These methods were introduced by Curtiss and Hirschfelder in Curtiss and Hirschfelder (1952). The Backward differentiation formulas with an order less than three are often used for stiff systems due to their property of being A-stable. We are interested in the second-order backward differentiation formula (BDF2). On an equidistant grid the BDF2 formula has the form:

2 $$ \frac{2}{3}x_{n+1}-2x_{n} + \frac{1}{2}x_{n-1} = hf_{n+1}(t_{n+1},x_{n+1}) $$

On a non-uniform grid, the BDF2 method can be formulated as:

3 $$ x_{n+1}=\alpha_{1}x_{n}+\alpha_{2}x_{n-1}+\beta h_{n+1}f(t_{n+1},x_{n+1}) $$

where

4 $$\begin{array}{@{}rcl@{}} &&\gamma_{n+1}=h_{n+1}/h_{n} \end{array} $$

5 $$\begin{array}{@{}rcl@{}} &&\alpha_{1}=1-\alpha_{2} \end{array} $$

6 $$\begin{array}{@{}rcl@{}} &&\alpha_{2} = -\gamma_{n+1}^{2}/(2\gamma_{n+1}+1) \end{array} $$

7 $$\begin{array}{@{}rcl@{}} &&\beta=(\gamma_{n+1}+1)/(2\gamma_{n+1}+1) \end{array} $$

#### Error control

To ensure that the exact solution is adequately approximated and the local error is within reasonable bounds we implement an error control mechanism of the coupled system during the simulation. Brocke et al. (2016) present an estimate of the local discretization error based on a component-wise estimation for the solution vector of the whole system at each discretization time step in a singlerate integration approach. The choice was justified by the theoretical background provided in Skelboe (2000). Analyzing the test case developed in Brocke et al. (2016) we noticed that it was the local discretization error of the fastest component that constrained the size of the discretization time step for the larger part of the simulation time. Bearing this in mind we developed an idea how a multirate integration can be achieved when the discretization error of one of the components dominates. We relax the necessity of a singlerate integration by allowing the faster component to have a step size controller independent from the slower component; whereas we constrain the step size controller of the slower component by taking the maximum discretization error between the local error and the maximum discretization error of the faster components calculated over the current step. This strategy is depicted by lines# 11- 14 of Algorithm 1.

Given a set of ordered components *S* = {*s*_*r*_...*s*_*q*_} in the fast-first organization strategy, the discretization error at $t^{n+1}_{r}$ of the current component *s*_*r*_ can be written in the form:

8 $$\begin{array}{@{}rcl@{}} \left| \epsilon^{n+1}_{r} \right|&=& \max\left\{\max_{i}\frac{\left|x^{n+1}_{r,i} - \hat{x}^{n+1}_{r,i}\right|}{relTOL_{r}\cdot \left|x^{n+1}_{r,i}\right| + absTOL_{r,i}} ,\right.\\&&\qquad\quad\left.\vphantom{\frac{\left|x^{n+1}_{r,i} - \hat{x}^{n+1}_{r,i}\right|}{relTOL_{r}\cdot \left|x^{n+1}_{r,i}\right| + absTOL_{r,i}}} \max_{m\in [k,k+j]}\left|\epsilon^{m}_{r+1}\right| \right\} \end{array} $$

The first argument in the braces corresponds to a component-wise calculation of the local discretization error of the *s*_*r*_ component. We use a predictor-corrector algorithm where $\hat {x}^{n+1}_{r}$ is a predicted solution and $x^{n+1}_{r}$ is an approximated solution at $t^{n+1}_{r}$. For the former we used a second order polynomial predictor. Details can be found in Brocke et al. (2016). *r* *e* *l* *T* *O* *L*_*r*_ and *a* *b* *s* *T* *O* *L*_*r*_ are input tolerance parameters. The second argument denotes the maximum discretization error of the faster component *s*_*r*+1_ over an integration interval $[t^{k}_{r+1},t^{k+j}_{r+1}]$. In synchronous communication between the components the interval is equal to the time step over $[{t^{n}_{r}},t^{n+1}_{r}]$ of the current component. The interval overlaps with the latter time step in an asynchronous communication as shown in Fig. 2.

### Test case

In the scope of interest are the models that are usually formulated on the cellular and subcellular levels of neuronal organization where the molecular events closely interact with the electrical ones and give rise to many levels of physiological change like synaptic plasticity and changes in dendritic excitability.

**Fig. 4:**
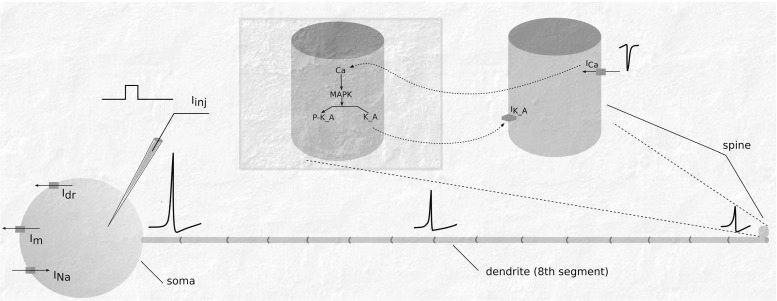
A schematic representation of the multiscale neuron assembly. The compartmental structure of the neuron is shown in terms of a soma, 15 segments of a dendrite and a spine. The spine segment is zoomed in for visual convenience. The MAPK model (biochemical component) is placed in the spine segment and is marked with a distinct rectangle. Communication signals between electrical and biochemical components are shown by the curved arrows. The response time of the MAPK model is in the range of tens of seconds (Bhalla 2011), while the response of the electrical model is the order of a microsecond

Here we exploit the multiscale test case used in Brocke et al. (2016). The test case was developed with two goals in mind: to keep complexity of the system within reasonable computational bounds, and to implement a moderately realistic behavior that will cover a class of problems among which synaptic and cellular plasticity are of particular interest. The electrical component of the system represented a multi-compartmental neuron with the minimal Hodgkin-Huxley type model for a regular-spiking neuron (Pospischil et al. 2008). During the simulations a pulse current protocol was applied to maintain the regular spiking behavior at approximately 2 Hz and the burst firing at approximately 100 Hz for 5 seconds of simulation time. The biochemical component of the system represented a reduced model of the Mitogen-Activated Protein Kinase (MAPK) signaling pathway previously used in the study of homeostatic regulation of excitability at the scale of a single synapse (Bhalla 2011). The biochemical component models the signaling events triggered by the electrical activity of the neuronal membrane. In particular, it is the calcium signal that feeds into a slowly integrating signaling pathway and initiates the chemical cascades. A schematic representation of the models and their interaction is shown in Fig. 4.

In the implementation of Brocke et al. (2016) the calcium current is transformed into a flux of calcium molecules and then communicated to the biochemical component. In this case the communication signal follows the fast dynamics of the electrical system.

**Fig. 5:**
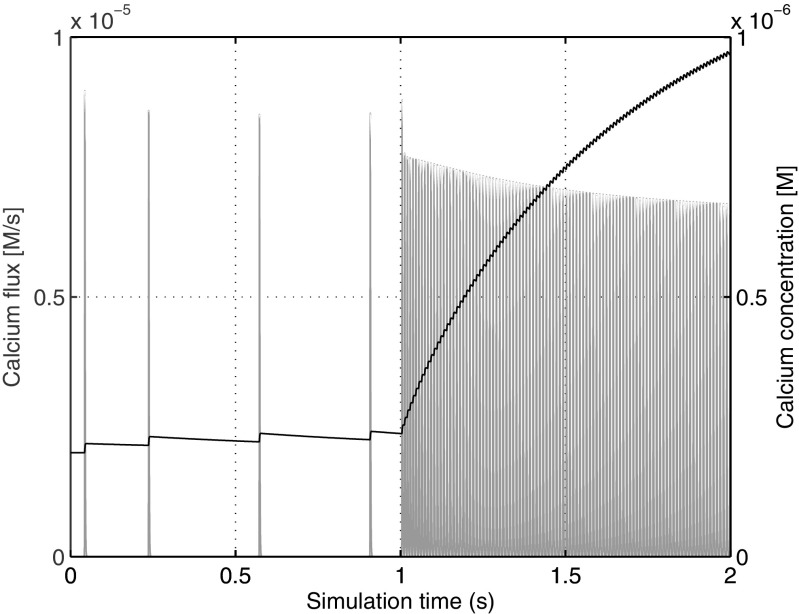
The communication signal from the electrical to biochemical component. Calcium flux (used in *TC-fast*) is shown in a *grey color* while calcium concentration (used in *TC-slow*) is shown in *black*. Calcium concentration exhibits a much smoother behavior

To investigate how the dynamics of the communication signal can influence the efficiency of a coupled integration, we introduce the following modifications to the test case. We solve calcium concentration on the electrical side and then communicate it to the biochemical component. The signal from the biochemical component to the electrical one, that is the fraction of active (non-phosphorylated) potassium channels in the spine, remains unchanged. We will refer to the original test case that has a fast changing communication signal, that is calcium flux, as *TC-fast*, and to the modified version with the slowly changing exchanged variable, that is calcium concentration, as *TC-slow*. In Fig. 5 we plot both communication signals for visual comparison: calcium flux and calcium concentration. It is clear that the dynamics of the calcium concentration is much slower than the dynamics of the flux. Here, microseconds are the characteristic time for the flux, and milliseconds for the concentration.

The morphological and physiological details of the test case *TC-slow* is given in Appendix A.

This modification of the communication signal leads to a *linear* dependence between the components in the problem (1):

9 $$\begin{array}{@{}rcl@{}} \frac{d}{dt}x_{bioch} &=&f_{bioch}(t,x_{bioch},g_{1}(x_{elec}))\\ \frac{d}{dt}x_{{elec}} &=&f_{{elec}}(t,g_{2}(x_{{bioch}}),x_{{elec}}), \end{array} $$

where *g*_1_ and *g*_2_ are the output functions from the electrical and the biochemical component respectively.

10 $$\begin{array}{@{}rcl@{}} g_{1}(x_{{elec}}) &=& x_{elec,i}\\ g_{2}(x_{{bioch}}) &=& C_{1}x_{{bioch},j} \end{array} $$

where *C*_1_ is a constant; the indices *i*,*j* correspond to positions of the variables in the corresponding solution vector *x* at time *t*. In particular, *i* is the calcium concentration in the spine [M] and *j* is the concentration of active (non-phosphorylated) potassium channels in the spine [M].

### Evaluation and implementation

In this paper we evaluate the proposed multirate algorithm using the multiscale test cases *TC-fast* and *TC-slow*. In order to gain understanding of the global error propagation in the coupled simulation, we should choose the most representative solutions from both electrical and biochemical components.

The phosphorylated MAPK (*P-MAPK*) and active potassium (*K_A*) molecules are the ones that bring about the bistability property of the biochemical system. We measure the accuracy by calculating the relative error of the solution at *T* = 2 *s* of the simulation time:

11 $$ e_{i}=\frac{|\check{x}_{T,i}-x_{T,i}|}{\check{x}_{T,i}}\cdot 100\quad[\%],  $$

where $\check {x}_{T,i}$ is the reference solution at time *T* and $\check {x}_{T,i}$ is the chosen solution at time *T* simulated with the analyzed method. In our simulations we choose to evaluate the voltage solution in the spine (*V*), calcium solution (*Ca*) and either potassium(*K_A*) or *P-MAPK* solutions in our test cases.

The test case, the algorithms and numerical methods were implemented in the MATLAB^®^ environment to attain a full control over numerical integration. The reference solution for each test case is acquired by solving both components as a single system using the *o* *d* *e*15*s* MATLAB^®;^ function with a tight value of the tolerance parameter (*t* *o* *l* = 10^−12^).

**Fig. 6:**
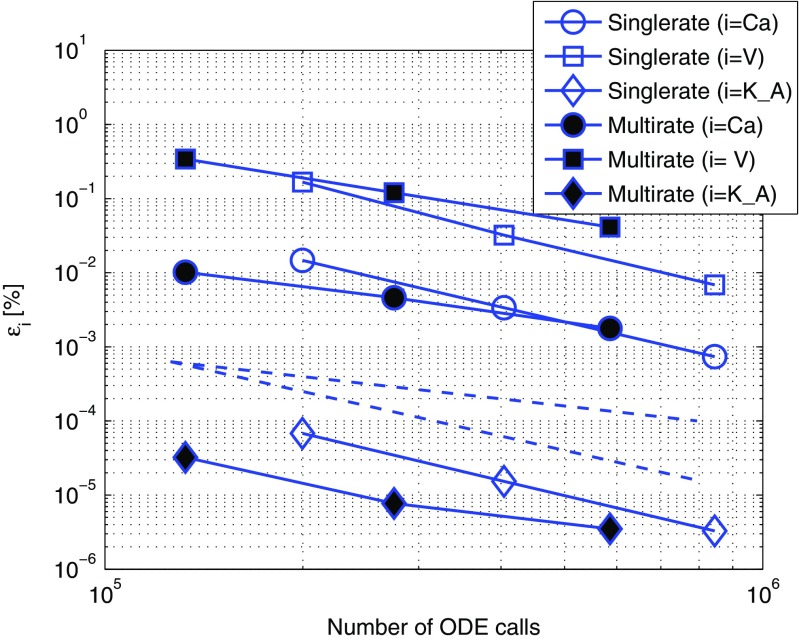
Efficiency comparison between single- and multirate approaches in the BDF2-BDF2 coupling method with the fast communication signal. The singlerate coupling method corresponds to the results presented in (Brocke et al. 2016). The datapoints in the figure correspond to *r* *e* *l* *T* *O* *L* = {10^−5^;10^−6^;10^−7^}. The *dashed lines* correspond to first- and second-order declines. The simulations were performed with the *TC-fast* test case that uses calcium flux as a communication signal between the electrical and biochemical component. The accuracy results of the singlerate integration using Gauss-Seidel organization where the electrical component was solved first is represented by hollow markers. The results of a multirate integration with Gauss-Seidel organization and the fast-first strategy are shown with filled markers. Exchanged variables were approximated by a second order polynomial (referred to as *Mode 3* in (Brocke et al. 2016))

**Fig. 7:**
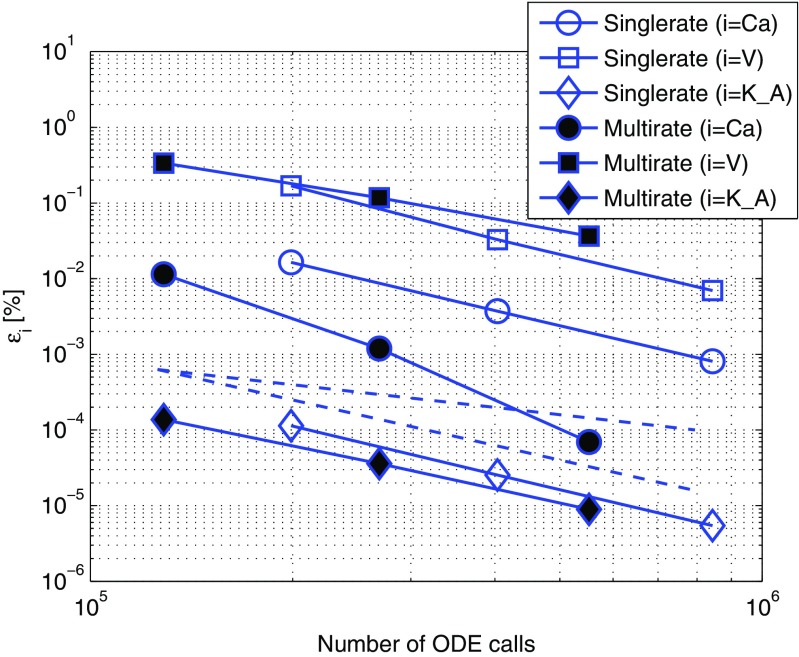
Efficiency comparison between single- and multirate approaches in the BDF2-BDF2 coupling method with the slow communication signal. The datapoints in the figure correspond to *r* *e* *l* *T* *O* *L* = {10^−5^;10^−6^;10^−7^}. The simulations were performed with the *TC-slow* test case which has a slow changing communication signal (calcium concentration) between the electrical and biochemical component. Otherwise conditions are as in Fig. 6. Hollow markers: singlerate integration with Gauss-Seidel organization, electrical component solved first. Filled markers: the results of the multirate integration with the fast-first strategy

**Fig. 8:**
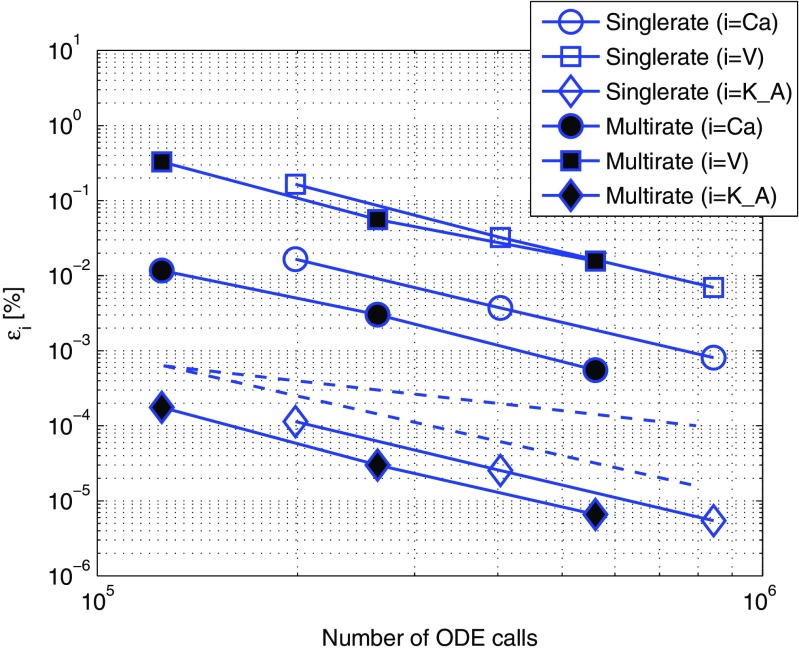
Efficiency comparison between single- and multirate approaches in the BDF2-BDF2 coupling method using slow-first strategy and a slow communication signal. The datapoints in the figure correspond to *r* *e* *l* *T* *O* *L* = {10^−5^;10^−6^;10^−7^}. The simulations were performed with the *TC-slow* test case. Gauss-Seidel organization with the biochemical component solved first and slow-first strategy were used in single- and multirate approaches, respectively

The sources containing the algorithms and model implementations as well as the datafiles to produce the Figs. 6–8 are available at https://github.com/ebrocke/multiscale.

## Results

Each figure in this section represents the measurements of the solution accuracy versus the number of ODE calls. We consider the number of ODE calls as a measure of the computational cost for the solver. We plot both first- and second-order declines in dashed lines in order to have visual estimates of the order of the coupling method. The singlerate coupling strategy in Brocke et al. (2016) showed a second order accuracy and is taken as a reference for evaluation of the multirate method in this paper. In addition, by comparing the curves vertically it is possible to estimate the accuracy of the solution for the same amount of computational cost. Looking at the curves horizontally, the computational costs for the same accuracy can be compared. Each curve is an interpolation on a set of datapoints marked with markers. Each datapoint corresponds to the specified tolerance parameter used in the simulation.

### Multirate improves efficiency but accuracy is lost with a fast communication signal

Second order accurate numerical solutions are expected using the BDF2-BDF2 coupling method since this was found with the *TC-fast* test case singlerate simulations in Brocke et al. (2016). To investigate the multirate algorithm’s performance we simulated the *TC-fast* test case with different values of the tolerance parameter. We plotted the accuracy measurements of the solutions versus the number of ODE calls in Fig. 6.

In the multirate simulations we observed a loss of order of accuracy in the *TC-fast* test case that is clearly noticeable while comparing the interpolated error behavior with the decline lines plotted in the figure. Presumably the dominant extrapolation error at each micro time step propagates to the slow component through the fast communication signal.

Comparing corresponding datapoints in Fig. 6, a large difference in the number of ODE calls between single- and multirate coupling approaches can be observed. For a detailed numerical overview of this difference we present the synopsis of the simulations in Table 1. The speed ups both with respect to the number of function calls and computation times in the multirate approach were substantial.

**Table 1:** Synopsis of the *TC-fast* test case simulations using single- and multirate approaches

relTol	micros	macros	speedup 1	speedup 2	# of switches
1e−5 (singlerate)	90529(4081)	–	–	–	–
1e−5 (multirate)	90543(4266)	23747(16)	1.5	3.4	26
1e−6 (singlerate)	194883(2915)	–	–	–	–
1e−6 (multirate)	193630(1822)	70244(74)	1.47	3.	81
1e−7 (singlerate)	417315(2211)	–	–	–	–
1e−7 (multirate)	416832(1922)	159361(89)	1.44	2.7	133

micros - the number of the micro time steps in case of the multirate or the total number of accepted steps otherwise; macros - the number of the macro time steps; speedup 1 - the speed up measurements based on the number of function calls; speedup 2 - the speed up measurements based on a wallclocktime; # of switches

- the number of times when the integration order of the components has been changed. The numbers in parenthesis denote the number of rejected steps during the simulation

### The extrapolation error influence in the fast-first strategy is reduced by a slow changing communication signal

Can we diminish the influence of the expected extrapolation error observed in the previous results? To answer this question we replaced the fast communication signal with the slow one as described in Section 2.3. Here, we simulate the *TC-slow* test case and compare single- and multirate coupling approaches in Fig. 7.

Comparing Figs. 6 and 7 we observed that the extrapolation error in the fast component (*i* = *V*, the voltage error lines) was not changed. However, the error in the slow component (*i* = *K*_*A*, the potassium error lines) was not only considerably smaller but, also, the second order accuracy was re-established. While the first observation was in accordance with our expectations due to required extrapolation in the fast-first strategy step, the latter was slightly surprising. We compared the order of accuracy of the communication signal (*i* = *C* *a*, the calcium error lines). The results showed that the accuracy of the communication signal, that is calcium concentration solved in the fast component, had been re-established to second order.

### Slow-first strategy restores the accuracy of the coupled multirate simulation

Our previous results showed that the second order of the BDF2-BDF2 coupling using the multirate integration approach was reduced to first order. The simulations were performed using the fast-first strategy where the variables communicated from the slow component had to be extrapolated at each micro time step. There, we suspected that an extrapolation error of the exchanged variables had the main contribution to the numerical approximation error at each discretization time step. After, instead, using a slow communication signal (the *TC-slow* test case) and verifying that the propagation of the approximation error can be diminished we applied the slow-first strategy. In that case an extrapolation of a slow varying signal may have less significant contribution to the discretization error of a slow component. Figure 8 confirms our expectations. Second order accuracy of the multirate algorithm was confirmed using the slow-first strategy and a slow communication signal between the components.

## Discussion

In this paper we presented a new multirate numerical integration algorithm based on the BDF2-BDF2 coupling method described in Brocke et al. (2016). The idea of multirate numerical integration is to let individual components be solved independently for a large integration time in order to gain efficiency by minimizing communication and computation operations. The proposed multirate algorithm showed computational speedups both with respect to the number of function calls and computation times in simulations of a given test case.

We presented and analyzed two organization strategies between the components, fast-first and slow-first. The simulations showed a strong dependence of the numerical accuracy of the coupled simulations on the dynamics of the communication signal between the components. We observed that in multirate simulations the second order of the BDF2-BDF2 coupling method was reduced to the first order in the fast-first strategy. We introduced a few modifications to our multiscale test case. In particular, we replaced the fast communication signal with its analogous slow changing variable. The results showed a strong numerical sensitivity of a slow system to the behavior of the communication signal. As can be seen in Fig. 7, the increased accuracy of the slow communication signal led to the re-establishment of the accuracy order of the slow component. Note, that a coupled method may have properties completely different from those of the individual components (Hanke 2017). It is known that the order of a method may depend on linearity and non-linearity of the system (Hairer et al. 1993). Considering that the slow variable had been defined by a linear ODE in comparison to its highly nonlinear definition in the initial test case, it might be one of the possible explanations for the re-establishment of the order in Fig. 7. In addition, we suspect that the stability domain of the coupled BDF2 method may change similar to what is observed in Hanke (2017). Some methods are known to have a reduced order if applied to very stiff problems (Hairer and Wanner 1996). It might happen that the smoothened variable drives the system away from stiffness thus changing the order. However, these ideas do not yet have any theoretical justification with respect to the coupled BDF2 methods.

The re-established order of the communication signal allowed us to employ a slow-first Gauss-Seidel organization strategy where the extrapolation error of the slow changing variable had a less severe impact on the accuracy of the coupled simulation thus making the slow-first organization strategy more favorable.

We decided to use calcium as a key communication signal between electrical and chemical components in the test case. From the perspective of cellular physiology it is clear that by far the most commonly reported and predominant signal for electrical to chemical coupling is indeed calcium. In our simulations we replaced the fast calcium flux communication signal with a smoother signal (calcium concentration). A few studies have estimated coupling through synaptic transmission via metabotropic G-protein coupled receptors (Bhalla and Iyengar 1999), but here flux calculations are not needed and instead the arrival of an action potential is typically treated as an instantaneous event that raises the activation of the receptor. Downstream events are slow (hundreds of ms) so this component of coupling is not likely to contribute significantly to the error. Neuronal simulation packages such as NEURON, GENESIS, MOOSE offer to solve chemical concentrations along with the fast electrical calculations. Here we represented calcium calculations by a phenomenological model of the calcium activity on the electrical side of the system. The results presented in the paper will remain valid unless a study addresses calcium behavior on a finer compartmentalization level. In this case a more detailed modeling of calcium and its interactions is required where calcium may not be viewed as a slow changing variable anymore.

An important biochemical aspect related to the chemical signal is diffusion. In spatially complex models, the chemical signals may require reaction-diffusion formulations. This introduces spatial discretization requirements for the chemical system and a further numerical integrator to solve the Partial Differential Equations (PDEs) for diffusion. Our current analysis does not address how to couple the chemical reaction calculations both to the chemical diffusion and electrical calculations.

The multirate algorithm developed in this paper is based on the BDF2-BDF2 coupling method. Solver implementations based on the BDF family of methods can be found in multiple scientific open source software packages which can easily be incorporated into existing simulation codes. For example, the NEURON simulation environment supports adaptive integration by means of the SUNDIALS CVODE suite (Serban and Hindmarsh 2005). The latter includes a variable-order variable-stepsize BDF-based method.

For purposes of evaluating the multirate algorithm we simulate the developed test case on a range of low tolerances. Our basic assumption is that the underlying system of ODEs is perfect, that is it depicts the real system with high fidelity. In reality, the knowledge of a system is usually constrained by some unknowns, for example, with regard to parameters. Thus it may turn out that it is not necessary to perform simulations with high levels of numerical precision. Therefore, once the method is designed well, other optimizations may be considered in order to achieve more efficient simulations. One possibility is to take different sources of error into consideration rather than to focus only on the numerical error. Rangan and Cai (2007) present a method that accounts for both *trajectory-wise* and *statistical* accuracies in simulations of large-scale integrate-and-fire neuronal networks. In neuronal network simulations it is usually more important to obtain accurate statistical properties of the network rather than the individual time course for each of the neurons. The emphasis of statistical properties in the work by Rangan and Cai (2007) has a close connection to uncertainty quantification that is often posed in a statistical manner. This analysis extends much beyond the scope of this paper but can be considered for further development of efficient coupling algorithms for electrical-chemical systems.

This paper presents a new multirate numerical integration algorithm developed with a particular interest in co-simulations of electrical-biochemical systems in neuroscience. The ideas and requirements of the implementation presented in the paper can be exploited in the development of the API in communication frameworks such as the MUlti-SImulation Coordinator tool (MUSIC) (Brocke and Djurfeldt 2011; Djurfeldt et al. 2010).

## Electronic supplementary material

Below is the link to the electronic supplementary material.

(PDF 260 KB)
