# Supplementary material for: Multirate method for co-simulation of electrical-chemical systems in multiscale modeling
Source: J Comput Neurosci. 2017 Apr 7;42(3):245–56. doi: 10.1007/s10827-017-0639-7 (PMC5403853; doi:10.1007/s10827-017-0639-7)
Supplement: Supplementary file 1 — (PDF 260 KB) [file 10827_2017_639_MOESM1_ESM.pdf]

Noname manuscript No.
(will be inserted by the editor)

Multirate Method for Co-simulation of Electrical-Chemical Systems in Multiscale Modeling

Ekaterina Brocke[1,2,3] · Mikael Djurfeldt[4,*] · Upinder S. Bhalla[2,*] · Jeanette Hellgren Kotaleski[1,5] · Michael Hanke[6]

Model Details and Mathematical Formulation of the System Components

Exchanged variables between the components are marked bold.

Electrical component

The electrical component of the modeled system is given by a system of equations (1).

$$\begin{aligned} C_i \frac{dV_i}{dt} &= \sum_{j \in \mathcal{N}_i} \frac{(V_j - V_i)}{R_{a_{ij}}} + \frac{(E_{m_i} - V_i)}{R_{m_i}} + \sum_{s \in \mathcal{M}_i} f_s(V_i, [S]_{in}, [S]_{out}) g_s(p_a, p_i) + I_{inj_i} \\ \frac{dp}{dt} &= \frac{p_\infty - p}{\tau} \end{aligned} \quad (1)$$

Ekaterina Brocke
ekaterina.brocke@scilifelab.se

Mikael Djurfeldt
mikael@djurfeldt.com

Upinder Singh Bhalla
bhalla@ncbs.res.in

Jeanette Hellgren Kotaleski
jeanette.h.kotaleski@scilifelab.se

Michael Hanke
hanke@kth.se

[1]Science for Life Laboratory, School of Computer Science and Communication, KTH Royal Institute of Technology, Stockholm, Sweden

[2]National Centre for Biological Sciences, Bangalore, India

[3]Manipal University, Manipal, India

[4]PDC Center for High-Performance Computing, KTH Royal Institute of Technology, Stockholm, Sweden

[5]Department of Neuroscience, Karolinska Institute, Stockholm, Sweden

[6]Department of Mathematics, School of Engineering Sciences, KTH Royal Institute of Technology, Stockholm, Sweden

* These authors contributed equally to this work

where $p \in \{m, n, l, r, h, s\}$, p_∞ is an open probability for that class of gate at equilibrium, τ is a time constant for p to reach equilibrium. If only the rate constants α and β of the gate are known, then p_∞ and τ can be calculated as shown in (2).

$$\begin{aligned} p_\infty &= \frac{\alpha}{\alpha+\beta} \\ \tau &= \frac{1}{\alpha+\beta} \end{aligned} \tag{2}$$

The rate constatns α_p and β_p at which closed gate p transits to an open state and at which open gate p transits to the closed state respectively are voltage dependent. Thus p_∞ and τ are voltage dependent as well. Formulas are given in Table 1.

Table 1. Gate functions.

Function	Reference
$\alpha_h = 128/\exp((-46 - V_i)/18)$<br>$\beta_h = 4000/(1 + \exp((-23 - V_i)/5))$	[1]
$\alpha_m = 320\,\mathrm{vtrap}^1((-50 - V_i), 4)$<br>$\beta_m = 280\,\mathrm{vtrap}((V_i + 23), 5)$	[1]
$\alpha_n = 32\,\mathrm{vtrap}((-48 - V_i), 5)$<br>$\beta_n = 500\exp(-53 - V_i)/40$	[1]
$l_\infty = 1/(1 + \exp((-V_i - 35)/10))$<br>$\tau_l = \tau_{max}/(3.3\exp((V_i + 35)/20) + \exp(-(V_i + 35)/20))$	[2]
$\alpha_s = 55\,\mathrm{vtrap}((-27 - V_i), 3.8)$<br>$\beta_s = 940\exp((-75 - V_i)/17)$	[3]
$\alpha_r = 0.457\exp((-13 - V_i)/50)$<br>$\beta_r = 6.5/(\exp((-V_i - 15)/28) + 1)$	[3]

Voltages are expected in [mV] units.

The functions f_s and g_s are an ion channel type dependent. The definition can be found in Table 2.

Table 2. Definition of the ion channels for each compartment.

Compartment name	Ion channel (s)	f_s	g_s
soma	Voltage-dependent sodium (Na)	$(E_{Na} - V_1)$	$\bar{g}_{Na} m^3 h$
	Delayed rectifier potassium	$(E_K - V_1)$	$\bar{g}_{K_{dr}} n^4$
	Slow non-inactivating potassium	$(E_K - V_1)$	$\bar{g}_{K_m} l$
spine	High threshold calcium	$(\frac{R(C+273.15)}{2F} log \frac{[Ca]_{out}}{[Ca]_{in}} - V_3)$	$\bar{g}_{Ca} r s^2$
	Transient potassium (K_A)	$(E_{K_A} - V_3)$	$\bar{g}_{K_A} \frac{[\mathbf{K\_A}]}{[\mathbf{K_{base}}]}$

1

```
function out = vtrap(x,y)
if (abs(x/y) < 1e-6)
    out = y(1-x/y/2);
else
    out = x/(exp(x/y)-1);
end
```

Calcium concentration $[Ca]_{in}$ in the spine is calculated on the electrical side:

$$\frac{d[Ca]_{in}}{dt} = -(f_{Ca}g_{Ca})/depth/F/2/10^{-10} + \frac{([Ca]_{out} - [Ca]_{in})}{\tau_{Ca}} \quad (3)$$

The membrane capacitance C of the compartment i and the axial resistance betwen the compartments i and j is given by (4) and (5) respectively.

$$C_i = C_M \cdot A_i \quad (4)$$

$$R_{a_{ij}} = \frac{R_{a_i} + R_{a_j}}{2}, \quad (5)$$

where $R_{a_i} = \frac{4.0 l_i R_A}{\pi d_i^2}$. The definitions and the values of the parameters can be found in Table 3 and Table 4.

Table 3. Geometric dimensions of the modeled neuron.

**Compartment name**	**Number of sub-compartments**	**Shape**	**Length (l_i) [μm]**	**Diameter (d_i) [μm]**	**Area (A_i)**
Soma	-	sphere	-	96	πd_1^2
Dendrite	15	cylinder	500	1	$\pi d_2 l_2$
Spine	-	cylinder	1	1	$\pi d_3^2 l_3/4$

Table 4. The description and parameter values in the electrical component.

**Parameter**	**Name**	**Value**	**Unit**
C_M	Specific membrane capacitance	0.01	F/m^2
R_A	Specific axial resistance	0.354	Ω·m
$E_{m_1} = E_{m_2} = E_{m_3}$	Membrane leakage potential	-0.07	V
g_{m_1}	Soma membrane conductance per area	1	S/m^2
g_{m_2}	Dendrite membrane conductance per area	6	S/m^2
g_{m_3}	Spine membrane condunctance per area	0.01	S/m^2
E_{Na}	Nernst equilibrium sodium potential	0.05	V
E_K	Nernst equilibrium potassium potential	-0.09	V
$\bar{g}_{Na}$	Maximum sodium conductance per area in the soma	500	S/m^2
$\bar{g}_{K_{dr}}$	Maximum delayed rectifier potassium conductance per area in the soma	50	S/m^2
$\bar{g}_{K_m}$	Maximum non-inactivating potassium conductance per area in the soma	0.7	S/m^2
$\bar{g}_{Ca}$	Maximum calcium conductance per area in the spine	30	S/m^2
$\bar{g}_{K_A}$	Maximum transient potassium conductance per area in the spine	34.5	S/m^2
τ_{max}	Time constant for adaptation [4]	0.8245	s
$[Ca]_{out}$	Concentration of Ca^{2+} in the extracellular fluid	2.0×10^{-3}	M
F	Faraday's constant	9.6485309×10^4	$C \cdot mol^{-1}$
C	Temperature in Celsius	36	°C
R	Universal gas constant	8.31441	$J \cdot K^{-1} \cdot mol^{-1}$
τ_{Ca}	Calcium time constant	800×10^{-3}	s
$depth$	Calculated as $volume/area$ of the spine	250	nm

The current is injected to the soma in a stepwise form as shown in Fig 1.

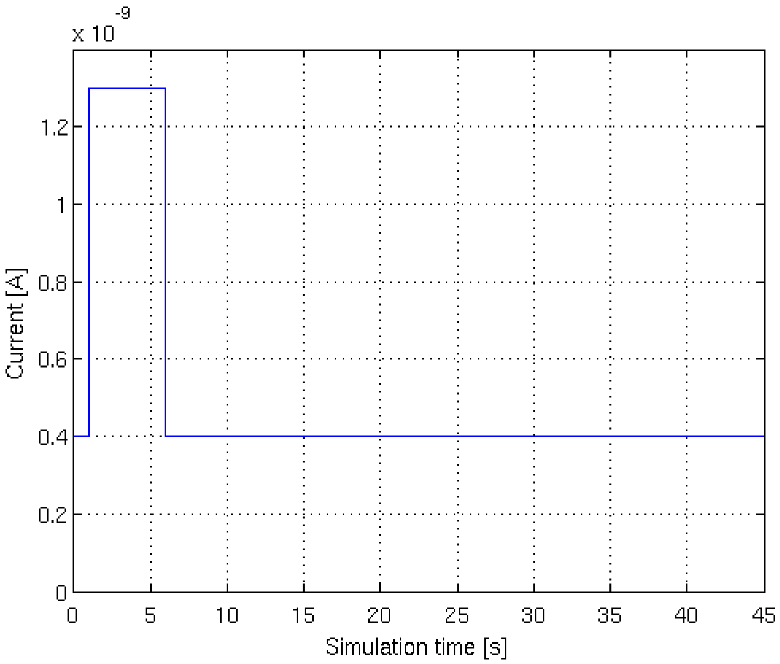

Fig 1. Soma stimulation protocol.

Biochemical component

The biochemical model is described by the set of chemical reaction rate equations represented in Table 5. Each molecule concentration then can solved with the ODE (6a)-(6r). The molecule index and its initial concentration value can be found in Table 6.

Table 5. The reaction scheme in the biochemical model and the rate values k_α, k_β and k_γ respectively.

	**Reaction**	k_α	k_β	k_γ
**1**	$2\,Ca + Raf \underset{k_\beta}{\overset{k_\alpha}{\rightleftharpoons}} Active\_Raf$	4e12	8.0	-
**2**	$Active\_Raf + MAPK \underset{k_\beta}{\overset{k_\alpha}{\rightleftharpoons}} Active\_Raf–MAPK \xrightarrow{k_\gamma} Active\_Raf + P\text{-}MAPK$	0.025090663e8	40.0	10.0
**3**	$Phosphotase + P\text{-}MAPK \underset{k_\beta}{\overset{k_\alpha}{\rightleftharpoons}} Phosphotase–P\text{-}MAPK \xrightarrow{k_\gamma} Phosphotase + MAPK$	0.501831326e8	0.4	0.1
**4**	$P\text{-}MAPK + K\_A \underset{k_\beta}{\overset{k_\alpha}{\rightleftharpoons}} P\text{-}MAPK–K\_A \xrightarrow{k_\gamma} P\text{-}MAPK + P\text{-}K\_A$	0.050184337e8	40.0	10.0
**5**	$P\text{-}K\_A \xrightarrow{k_\alpha} K\_A$	0.05	-	-
**6**	$PKC + 2\,AA \underset{k_\beta}{\overset{k_\alpha}{\rightleftharpoons}} Active\_PKC$	1e12	2.0	-
**7**	$AA \underset{k_\beta}{\overset{k_\alpha}{\rightleftharpoons}} APC$	0.2	0.01	-
**8**	$P\text{-}MAPK + APC \underset{k_\beta}{\overset{k_\alpha}{\rightleftharpoons}} P\text{-}MAPK–APC \xrightarrow{k_\gamma} P\text{-}MAPK + AA$	0.250918674e8	20.0	5.0
**9**	$Active\_PKC + MAPK \underset{k_\beta}{\overset{k_\alpha}{\rightleftharpoons}} Active\_PKC–MAPK \xrightarrow{k_\gamma} Active\_PKC + P\text{-}MAPK$	0.050184337e8	4.0	1.0
**10**	$PMCA + Ca \underset{k_\beta}{\overset{k_\alpha}{\rightleftharpoons}} PMCA–Ca \xrightarrow{k_\gamma} PMCA$	0.06e7	7.0	5.0

The reaction rate constants are given in [1/(M·s)] units.

$$\frac{d[S_2]}{dt} = -k_{\alpha_1}[\mathbf{Ca}]^2[S_2] + k_{\beta_1}[S_3] \tag{6a}$$

$$\frac{d[S_3]}{dt} = k_{\alpha_1}[\mathbf{Ca}]^2[S_2] - k_{\beta_1}[S_3] - k_{\alpha_2}[S_3][S_4] + (k_{\beta_2} + k_{\gamma_2})[S_5] \tag{6b}$$

$$\frac{d[S_4]}{dt} = -k_{\alpha_2}[S_3][S_4] + k_{\beta_2}[S_5] + k_{\gamma_3}[S_8] - k_{\alpha_9}[S_4][S_{13}] + k_{\beta_9}[S_{16}] \tag{6c}$$

$$\frac{d[S_5]}{dt} = k_{\alpha_2}[S_3][S_4] - (k_{\beta_2} + k_{\gamma_2})[S_5] \tag{6d}$$

$$\begin{aligned}\frac{d[S_6]}{dt} &= k_{\gamma_2}[S_5] - k_{\alpha_3}[S_6][S_7] + k_{\beta_3}[S_8] - k_{\alpha_4}[S_6][S_9] + (k_{\beta_4} + k_{\gamma_4})[S_{10}] \\ &\quad - k_{\alpha_8}[S_6][APC] + (k_{\beta_8} + k_{\gamma_8})[S_{15}] + k_{\gamma_9}[S_{16}]\end{aligned} \tag{6e}$$

$$\frac{d[S_7]}{dt} = -k_{\alpha_3}[S_6][S_7] + (k_{\beta_3} + k_{\gamma_3})[S_8] \tag{6f}$$

$$\frac{d[S_8]}{dt} = k_{\alpha_3}[S_6][S_7] - (k_{\beta_3} + k_{\gamma_3})[S_8] \tag{6g}$$

$$\frac{d[S_9]}{dt} = -k_{\alpha_4}[S_9][S_6] + k_{\beta_4}[S_{10}] + k_{\alpha_5}[S_{11}] \tag{6h}$$

$$\frac{d[S_{10}]}{dt} = k_{\alpha_4}[S_9][S_6] - (k_{\beta_4} + k_{\gamma_4})[S_{10}] \tag{6i}$$

$$\frac{d[S_{11}]}{dt} = k_{\gamma_4}[S_{10}] - k_{\alpha_5}[S_{11}] \tag{6j}$$

$$\frac{d[S_{12}]}{dt} = -k_{\alpha_6}[S_{12}][S_{14}] + k_{\alpha_6}[S_{13}] \tag{6k}$$

$$\frac{d[S_{13}]}{dt} = k_{\alpha_6}[S_{14}]^2[S_{12}] - k_{\beta_6}[S_{13}] - k_{\alpha_9}[S_4][S_{13}] + (k_{\beta_9} + k_{\gamma_9})[S_{16}] \tag{6l}$$

$$\frac{d[S_{14}]}{dt} = -2k_{\alpha_6}[S_{14}]^2[S_{12}] + 2k_{\beta_6}[S_{13}] - k_{\alpha_7}[S_{14}] + k_{\beta_7}[APC] + k_{\gamma_8}[S_{15}] \tag{6m}$$

$$\frac{d[S_{15}]}{dt} = k_{\alpha_8}[S_6][APC] - (k_{\beta_8} + k_{\gamma_8})[S_{15}] \tag{6n}$$

$$\frac{d[S_{16}]}{dt} = k_{\alpha_9}[S_4][S_{13}] - (k_{\beta_9} + k_{\gamma_9})[S_{16}] \tag{6o}$$

$$\frac{d[S_{17}]}{dt} = -k_{\alpha_{10}}[\mathbf{Ca}][S_{17}] + (k_{\beta_{10}} + k\gamma 10)[S_{18}] \tag{6p}$$

$$\frac{d[S_{18}]}{dt} = k_{\alpha_{10}}[\mathbf{Ca}][S_{17}] - (k_{\beta_{10}} + k_{\gamma_{10}}[S_{18}] \tag{6q}$$

Table 6. Initial values of the molecule concentrations.

Molecule (S_i)		Initial concentration [M]
**2**	Raf	1e-6
**3**	Active_Raf	0.0
**4**	MAPK	1e-6
**5**	Active_Raf–MAPK	0.0
**6**	P-MAPK	0.0
**7**	Phosphatase	0.5e-6
**8**	Phosphotase–P-MAPK	0.0
**9**	K_A	1e-6
**10**	P-MAPK–K_A	0.0
**11**	P-K_A	0.0
**12**	PKC	1e-6
**13**	Active_PKC	0.0
**14**	AA	0.0
**15**	P-MAPK–APC	0.0
**16**	Active_PKC–MAPK	0.0
**17**	PMCA	4.122815331814883e-06
**18**	PMCA–Ca	7.928491022720928e-07
**19**	APC	1e-6

References

1. Traub RD, Miles R (1991) Neuronal networks of the hippocampus, volume 777. Cambridge University Press.
2. Yamada WM, Koch C, Adams PR (1989) Multiple channels and calcium dynamics. In: Methods in neuronal modeling. MIT press, pp. 97–133.
3. Kay A, Wong R (1987) Calcium current activation kinetics in isolated pyramidal neurones of the ca1 region of the mature guinea-pig hippocampus. The Journal of Physiology 392: 603–616.
4. Pospischil M, Toledo-Rodriguez M, Monier C, Piwkowska Z, Bal T, et al. (2008) Minimal hodgkin–huxley type models for different classes of cortical and thalamic neurons. Biological cybernetics 99: 427–441.
